# Pathophysiology, diagnosis and management of cerebral venous thrombosis: A comprehensive review

**DOI:** 10.1097/MD.0000000000036366

**Published:** 2023-12-01

**Authors:** Redoy Ranjan, Gie Ken-Dror, Pankaj Sharma

**Affiliations:** a Department of Cardiac Surgery, Bangabandhu Sheikh Mujib Medical University, Dhaka, Bangladesh; b Institute of Cardiovascular Research, Royal Holloway University of London (ICR2UL), Egham Hill, Greater London, United Kingdom; c Department of Clinical Neurology, Imperial College London Healthcare NHS Trust, London, United Kingdom.

**Keywords:** cerebral venous thrombosis, CVT, diagnosis, outcome, treatment

## Abstract

Cerebral venous thrombosis is a rare cause of stroke in young mostly female adults which is frequently overlooked due to its variable clinical and radiological presentation. This review summarizes current knowledge on it risk factors, management and outcome in adults and highlights areas for future research. Females are 3 times more commonly affected and are significantly younger than males. The presenting symptoms can range from headache to loss of consciousness. However, the often-nebulous nature of symptoms can make the diagnosis challenging. Magnetic resonance imaging with venography is often the diagnostic imaging of choice. While unfractionated or low molecular-weight heparin is the mainstay of treatment, endovascular intervention with thrombolysis or thrombectomy and decompressive craniectomy may be required depending on clinical status. Nevertheless, approximately 80% of patients have a good recovery but mortality rates of −5% to 10% are not uncommon. Diagnosing cerebral venous thrombosis can be challenging but with vigilance and expert care patients have the best chance of a good clinical outcome.

## 1. Introduction

Cerebral venous thrombosis (CVT) is a relatively rare condition that comprises approximately 0.5% to 1% of all stroke and is associated with an increased mortality rate.^[1,2]^ It is a multifactorial disease, with variable symptoms making immediate diagnosis challenging.^[2–4]^ The clinical presentation can be divided into 3 subcategories depending on the duration of onset: Acute ≤ 48 hours; Subacute > 48 hours to ≤ 30 days; Chronic ≥ 1-month forms^[5]^ of which the subacute presentation is the most common form which constituting almost half of all cases, while the chronic form is less frequent.^[5–8]^ Over the last few decades, the incidence of CVT has increased 10-fold due to better recognition and improved availability of advanced imaging modalities.^[4–6]^ Moreover, the increased incidence is found among younger adults, especially reproductive-age women (female-to-male ratio was 3:1) in low-income countries, probably associated with pregnancy, puerperium, and oral contraceptives.^[5,9–14]^ The risk factors for cerebral venous thrombosis are presented in Table 1.

**Table 1:** Risk factors of cerebral venous thrombosis.^[5,12,14–36]^

Prothrombotic states	Infection (12%)^[5,14,26–28]^
Hereditary conditions (34–41%)^[5,12,14–18]^	1. ENT and face infection (8.2–11%) 2. Systemic infectious diseases (4.3%) 3. Meningitis (2.1%)
1. Prothrombin G20210A mutation (9–21%) 2. Factor V Leiden mutation (9–13%) 3. MTHFR mutation (4.5%) 4. Antithrombin deficiency (3%) 5. Protein C deficiency (2–5%) 6. Protein S deficiency (2–3%)	
	Mechanical causes^[5,12,27–30]^
	1. Lumbar puncture (1.9%) 2. Head trauma (1.1%) 3. Jugular vein catheterization (1%) 4. Neurosurgical procedures (0.6%) 5. Trauma to cerebral sinuses
Acquired conditions (15.7%)^[5,12,14,17–19]^	
1. Pregnancy and puerperium (11–59%) 2. Antiphospholipid antibody syndrome (6–17%) 3. Nephrotic syndrome (0.6–1%) 4. Hyperhomocysteinemia	
	Malignancy (7.4%)^[30–32]^
	1. CNS tumors 2. Systemic malignancies 3. Myeloproliferative neoplasms
Haematology^[5,12,14,20]^	Drugs^[5,12,14,30,33,34]^
1. Severe anemia (9–27%) 2. Polycythaemia 3. Thrombotic thrombocytopenic purpura 4. Heparin-induced thrombocytopenia	1. Oral contraceptives (54–71%) 2. Hormone replacement therapy (4.3%) 3. Cytotoxic drugs (0.8%) 4. Intravenous immunoglobulin 5. Steroids
Autoimmune and Inflammatory diseases^[5,14,21–25]^	Miscellaneous^[12,14,30,35,36]^
1. Inflammatory bowel disease (1.6–3%) 2. Systemic lupus erythematosus (1%) 3. Behçet’s disease (1%) 4. Sarcoidosis (<1%) 5. Thyrotoxicosis (1.7%) 6. COVID-19 vaccine (<1%)	1. Obesity (23%) 2. Dehydration (1.9%) 3. Dural A-V fistulae (1.6%) 4. Arteriovenous malformations (0.2%) 5. No identifiable reasons (12.5%)

Methylenetetrahydrofolate Reductase (MTHFR); ENT- Ear, Nose, and Throat.

Percentage (%) denotes the prevalence of the risk factors. The data expressed in the table were obtained from original research works and review literature.^[5,12,14–36]^

**Figure 1.**
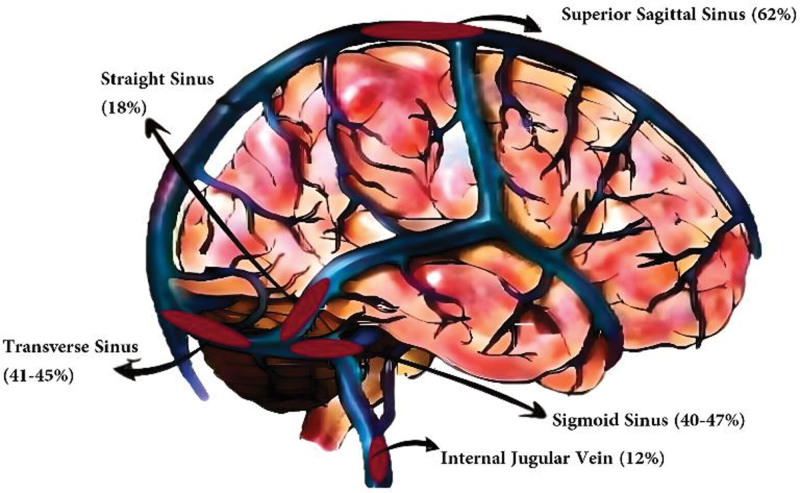
Anatomy of Dural venous sinus with distribution of CVT in percentage (Ferro et al^[5]^). CVT = cerebral venous thrombosis.

The International Study on Cerebral Vein and Dural Sinus Thrombosis documented the occurrence of CVT in different venous sinuses: superior sagittal sinus (62%), transverse sinus (41–45%), straight sinus (18%), cortical veins (17.1%), jugular veins (12%), a vein of Galen, and internal cerebral vein (11%) (Fig. 1).^[5]^ Furthermore, in recent studies authors also reported higher incidence of superior sagittal sinus (SSS) involvement 65%,^[8]^ 51%,^[37]^ and 45%^[38]^ cases and transverse sinus was 60.5%,^[8]^ 56%,^[37]^ and 62%^[38]^ patients. Authors also reported multiple venous sinus involvement in 71.2%,^[8]^ and 46%^[37]^ cases. The superficial venous circulation has numerous anastomoses and collateral circulation with variation in the course, which may explain the better prognosis of CVT involving the superficial venous system.^[7–12]^ However, the deep venous system is usually consistent and visible at angiography; thus, thrombosis in the deep venous sinus can be diagnosed easily.^[2–6,8–12]^

We searched electronic databases, especially MEDLINE, EMBASE, CINAHL, and Web of Science collection up to July 2023 to search literature on the epidemiology, clinical features, diagnostic modalities, treatment protocols, and prognosis of cerebral venous thrombosis utilizing medical subject headings terms and Boolean operators to combine search terms. This literature review aims to summarize the current knowledge on the epidemiology, pathophysiology and management of adult CVT.

## 2. Pathophysiology

**Figure 2.**
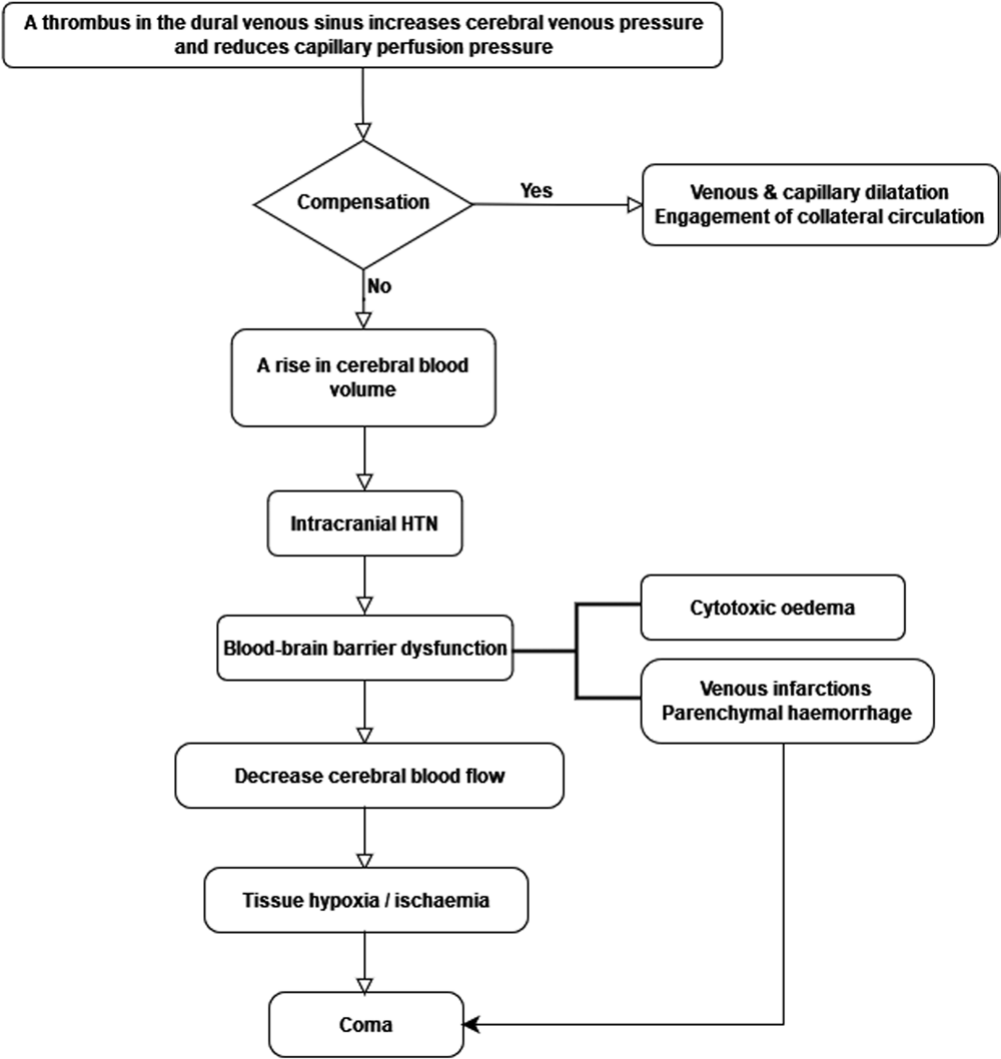
The flowchart illustrates the pathophysiological changes in CVT. The data for constructing this flowchart were obtained from the original studies evaluating the adult CVT population.^[2,3,10,11,14,37]^ CVT = cerebral venous thrombosis.

The entire pathophysiology has not been experimentally proven, but CVT may present as either of the following 4 distinct clinical syndromes: Intracranial hypertension; Focal neurological syndrome; Diffuse encephalopathy, and; Cavernous sinus syndrome.^[2–4,6]^ The pathophysiological changes in CVT evolve slowly over hours or days and can progress sufficiently for weeks to cause signs and symptoms of CVT. Cerebral vein thrombosis increases venous pressure and reduces capillary perfusion pressure, leading to a rise in cerebral blood volume; ultimately, patients develop intracranial hypertension.^[8–12]^ However, cortical collateral circulation is engaged, but intracranial hypertension subsequently leads to disruption of the blood-brain barrier and the development of vasogenic edema. This pathophysiology causes failure of the sodium-potassium ATPase dependent pump, an indirect regulator of intracellular water volume results in cytotoxic edema development.^[3,10–14,37]^ Figure 2 depicts the pathophysiological changes in CVT.

Superficial cortical veins drain into the SSS against the blood flow within the sinus, resulting in blood turbulence which is further aggravated by the existing fibrous septa at the inferior angle of the sinus. This is the most acceptable explanation of the higher prevalence of thrombosis in SSS.^[3–6,14]^ Furthermore, in addition to draining the cerebral hemisphere, the SSS and other dural venous sinuses also drain blood from diploic, meningeal and emissary veins. This explains the relationship between the occurrence of CVT following infective pathologies in their draining areas, For example, cavernous sinus thrombosis in facial infections, lateral sinus thrombosis in chronic otitis media and sagittal sinus thrombosis in scalp infections.^[3,12–14,37]^

The dural venous sinuses contain most of the arachnoid villi and granulations, especially in the SSS, responsible for cerebrospinal fluid absorption. So, thrombosis of the dural venous sinus causes blockage of villi and granulations and prevention of cerebrospinal fluid absorption, which eventually leads to intracranial hypertension and papilloedema provoked coma and mortality.^[14,37–40]^

## 3. Clinical presentations

The clinical presentation of CVT is often vague and largely depends on the site and extent of the lesion, age of onset, and associated comorbidities.^[8–12]^ CVT patients may present with a constellation of symptoms, which broadly categorize either as isolated such as intracranial hypertension (ICH) and a focal brain lesion, or a combination of both based on the extent of ICH and brain parenchymal lesion.^[15,16,37–40]^ However, about 40% of patients present with acute stroke-like syndrome within 48 hours of onset, and acute or subacute headache is the most common clinical presentation of CVT, often with a normal neurological finding. The most common clinical presentations are signs of intracranial hypertension and parenchymal drainage: headache (70–90%), seizure (30–40%), papilloedema (30–60%), focal neurological deficits (30–50%), aphasia (15–20%), altered level of consciousness (15–25%), coma (5–15%), and rarely movement disorder.^[16–18]^

The clinical symptoms corresponding to each type of dural venous sinus thrombosis or overviewed in Table 2. Physicians should be alert for CVT if a patient presents with the following potential symptoms:^[16,18,29,42,43]^

**Table 2:** Clinical presentations according to the affected dural venous sinuses.^[2–6,11–14,18,29,30,37–39,41–43]^

Site of CVT	Clinical presentation
Superior sagittal sinus (39–62%)	Cranial nerve palsies and intracranial hypertension lead to common symptoms: 1.Headache, nausea, vomiting 2.Blurred vision, occasionally loss of vision 3.Seizures 4.Aphasia, hemianopia 5.Hemisensory loss and/or hemiparesis 6.Rarely, isolated psychiatric symptoms
Transverse sinus (44–73%)	Isolated TS involvement without infarction: 1.Asymptomatic or 2.Headache, seizures Left TS involvement with venous infarction: Aphasia. Involvement of contiguous sinuses: 1.Intracranial hypertension 2.Cranial nerve IX–XXI palsies
Sigmoid sinus (40–47%)	1.Pain in the mastoid region 2.Cranial nerve VI–VIII palsies
Deep venous system (10.9%)	1.Diminished level of consciousness, or coma 2.Diffuse encephalopathy 3.Bilateral or fluctuating motor deficits
Cortical veins (3.7–17.1%)	Focal neurological deficits and seizures
Cavernous sinus (1.3–1.7%)	Headache, fever and ocular signs (ocular pain, chemosis, proptosis, ocular nerve palsy)
Inferior sagittal sinus	Motor deficits, seizures
Straight sinus	Motor deficits, mental status changes
Internal jugular vein	Neck pain, tinnitus, and cranial nerve palsies

The data for the construction of this table were obtained from reviews and original studies that evaluate clinical presentation and Dural venous sinuses involvement in the adult CVT population.^[2–6,11–14,18,29,30,37–39,41–43]^

CVT = cerebral venous thrombosis, TS = transverse sinus.

1. Headache in a young woman who recently started taking oral contraceptive pills or in a woman of the third trimester of pregnancy.
2. Persistent atypical headache in young adults.
3. Stroke of unknown etiology.
4. Haemorrhagic infarcts with abnormal cerebral vasculature or multiple hemorrhagic infarcts.
5. Eye symptoms following a recent attack of sinusitis.
6. New onset of seizures and focal neurological signs.
7. Altered level of consciousness.

## 4. Diagnosis

### 4.1. Overview of diagnostic modalities

The diagnosis of CVT is based on a high degree of clinical suspicion confirmed by either computed tomography (CT) or magnetic resonance imaging (MRI) with contrast-enhanced venography to demonstrate venous sinus thrombosis.^[5,14,37–40]^ The radiological findings of CVT can be direct visualization of venous sinus without blood flow; or maybe ischemic changes associated with the venous outflow obstruction.^[3,11,18]^ There is no specific laboratory test that can positively exclude CVT in the acute phase of the disease, and blood tests are performed to evaluate coagulation abnormalities like an underlying hypercoagulable state, systemic infection, or an inflammatory process. Furthermore, screening for potential prothrombotic conditions that may predispose to CVT is recommended.^[6,8,12,27–30]^ Details of the radiological findings of adult CVT are illustrated in Table 3.

**Table 3:** At a glance merits and demerits of CT, MRI, and DSA techniques.^[3,5,11,12,27,44–50]^

Techniques	Traits	Description
CT Venography	Advantages	1.Good visualization of major venous sinuses 2.Simple, less time consuming, and less motion artifacts 3.Useful in claustrophobic patients, pacemaker, or defibrillator.
	Disadvantages	1.Ionizing radiation exposure 2.Diabetes, and CKD patients may develop contrast nephropathy. 3.Poor resolution for small parenchymal lesion.
	Sensitivity and specificity	1.CT and CTV has 95% sensitivity and 91% specificity. 2.Based on the lesion, overall accuracy is 90% to 100%
	Typical findings	1.Hyperdensity and lack of flow in thrombosed sinuses 2.Dense triangle sign, empty delta sign and Cord sign
MR Venography	Advantages	1.No radiation exposure and good delineation of brain parenchyma. 2.Identify both of cortical and deep venous thrombosis. 3.Early ischemic changes can be detected.
	Disadvantages	1.Time consuming, unavailability and produce motion artifacts. 2.Unavailable for claustrophobic patients, and pacemaker. 3.Risk of gadolinium-induced nephrogenic systemic fibrosis
	Sensitivity and specificity	1.Not known; however, MRV with echoplanar T2 susceptibility-weighted image are considered as the most sensitive sequences.
	Typical findings	1.≤1 wk: Isointense in T1 and hypointense in T2W images. 2.Up to 2 weeks: Hyperintense on T1 and T2W images 3.>2 wk: Variable appearances; Hypointense in GRE and SWI images; Hyperintensity in DWI enhancement venous wall, and lack of flow in thrombosed sinuses.
DSA	Advantages	1.Precise dynamic information on collateral venous system. 2.Only performed when planned for an endovascular intervention.
	Disadvantages	1.Invasive procedure with associated procedural risks. 2.Skilled person required. 3.Usually, unavailable outside of tertiary hospital.
	Sensitivity and specificity	1.Not clearly known
	Typical findings	1.Absence of sinus opacification. 2.Venous congestion with dilated cortical, scalp, and facial veins. 3.Reversal of the flow and enlarged collateral venous drainage.

AV = arteriovenous, CT = computed tomography, CKD = Chronic kidney disease, CVT = Cerebral venous thrombosis, CTV = CT venography, DSA = digital subtraction angiography, MRI = magnetic resonance imaging, MRV = magnetic resonance venography, TOF = time-of flight.

### 4.2. CT scan and CT venography

Prompt investigation with an unenhanced CT scan of the brain is the noninvasive imaging method of choice when CVT is clinically suspected. Acute CVT may demonstrate an elongated hyper-attenuating clot known as a “cord sign,” which may persist for 2 weeks and then become isodense to brain parenchyma.^[18,40]^ Generally, a non-contrast CT scan produces an indirect sign that includes the early and late signs of venous ischemia known as sulcal effacement and diffuse parenchymal edema, ventricular effacement, or diminished differentiation between gray and white matter. However, a cerebral infarct not following a typical arterial territory, involving only a subcortical area, multiple unilateral and bilateral lesions with or without hemorrhagic changes should raise a concern about the venous origin.^[3,5,12]^ Further, a cerebral infarct comprising multiple arterial territories should raise concerns about potential venous pathology, particularly CVT.^[5,12,27]^

CT venography (CTV) is particularly useful in acute and emergency cases and can be utilized as the initial test for assessing the patency of the deep and cortical venous system in a comatose or uncooperative patient.^[8–11,18]^ The most frequent findings on CTV is vascular filling defects and an “empty delta sign” when the superior sagittal sinus is involved.^[44,45]^ However, an artifact from dense cortical bones significantly reduces the diagnostic accuracy of the CT venography, and also, arachnoid granulations may protrude into the venous sinuses, mimicking filling defects by thrombus, which is another potential disadvantage of CTV imaging.^[44–47]^ In infants, a false dense clot sign may result from the relatively high density of the blood in the sagittal sinus, and a false, empty delta sign may cause hyperdense empyema.^[5–10,48]^ Occasionally, engorged and dilated venous malformations produce a hyperdense lesion on unenhanced CT and demonstrate a characteristic linear enhancing focus converging on a single dilated vein known as “caput medusa” or “candelabra” appearance on CT venography.^[3,5–12,18,44]^

### 4.3. MRI and MR venography

Conventional T1 and T2 weighted MRI is more sensitive than an unenhanced CT scan to diagnose a case of CVT.^[45–47]^ On standard sequences, the early signs include the absence of a typical venous flow pattern and abnormal signal within the dural venous sinus. A brief description of the evolution of thrombus signal intensity caused by the paramagnetic effects of hemoglobin degradation products is provided in Table 3.

Magnetic resonance venography (MRV) is helpful in either acute or subacute and emergency or ambulatory cases and to confirm suspected cases of deep venous thrombosis where CT venography was inconclusive or normal.^[5,7–12,40,44,45]^ Contrast-enhanced MRV offers improved visualization of the cerebral venous system and is unlikely to be affected by complex blood flow.^[8,18,42]^ However, in MRI venography, aplasia and hypoplasia of the transverse sinus can be mistaken. There is also the chance of signal loss due to in-plane flow, and hyperintense thrombi can mimic patent sinus during time-of-flight angiography. Nonetheless, both MR venography and CT venography are adequate for CVT diagnosis, but MRV has higher diagnostic accuracy for the visualization of brain parenchymal lesions.^[6,41–45]^

### 4.4. Digital subtraction angiography (DSA)

Although DSA is considered a gold standard technique it usually only performed in the presence of either unclear CTV and MRV imaging or when endovascular intervention is planned because of its associated risk.^[29,44–49]^ Generally, there are filling defects in the dural venous sinuses or cortical veins, delayed venous drainage, and dilated collateral circulation. There may also be an abrupt cutoff of cortical veins with surrounding tortuous and dilated “corkscrew” collateral circulation.^[5,18,29]^ Furthermore, DSA can identify vascular aneurysm and dural arteriovenous fistula, which might cause the formation of a false “corkscrew” sign due to sluggish venous drainage and vascular congestion.^[50]^ Nevertheless, DSA has a unique ability to measure venous pressure and pressure > 10 mm H_2_O indicates a probability of parenchymal damage, which carries significant value for treatment outcome.^[41,45]^

## 5. Treatment and guidelines

### 5.1. Overview of treatment protocols

**Figure 3.**
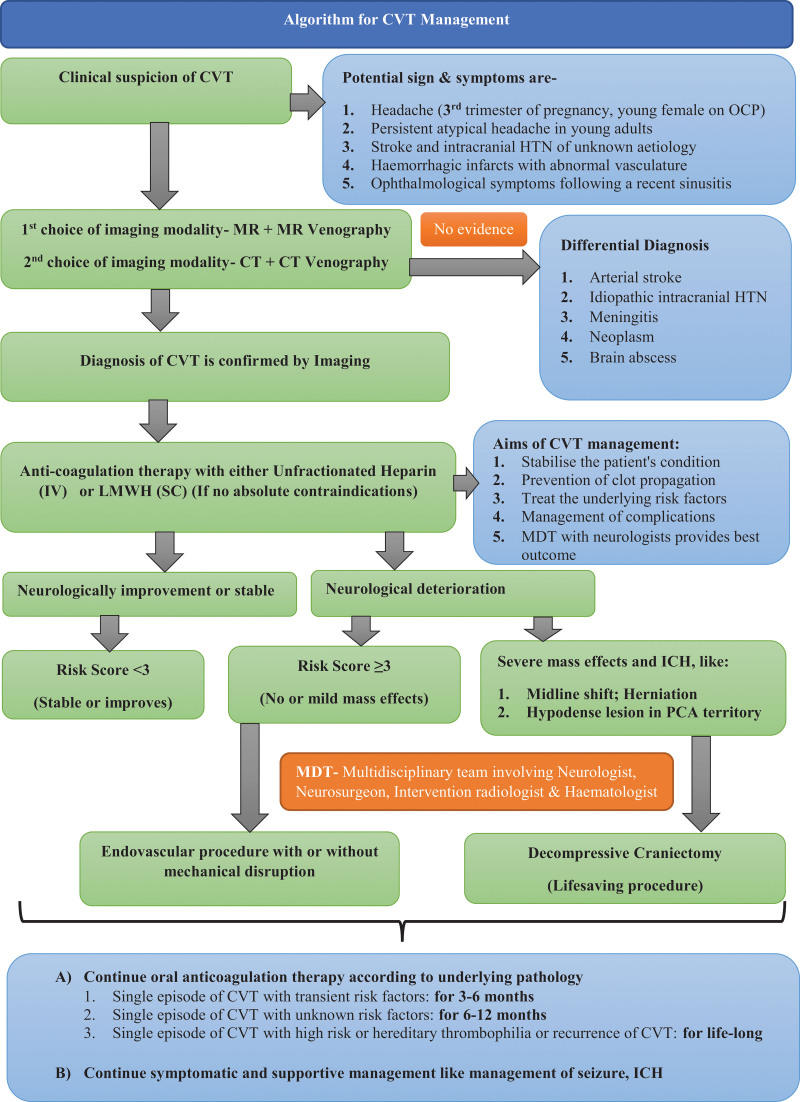
Algorithm of cerebral venous thrombosis management. The management algorithm of adult CVT was based on the published review literature and original studies that focus on the treatment outcome.^[5,11,14,18,41,49–56]^ CVT = cerebral venous thrombosis.

Prompt diagnosis to identify and treat the associated factors, initiate anticoagulation therapy, and manage ICH should maximize the chance of a favorable outcome.^[18,39,41]^ The management algorithm of CVT is illustrated in Figure 3.

### 5.2. Anticoagulation therapy

In 2011, American Heart Association-American Stroke Association guidelines proposed using full-dose unfractionated or low molecular weight heparin, followed by oral anticoagulant warfarin and acetazolamide.^[11]^ Furthermore, in the absence of significantly powered evidence from anticoagulant therapy trials, European Stroke Organization guidelines from 2017 recommend using low molecular weight heparin except heparin-induced thrombocytopenia, or vaccine-induced immune thrombotic thrombocytopenia, decompressive craniectomy if ICH is present, and anticonvulsant medication in seizures.^[18]^ However, neither European Stroke Organization nor American Heart Association-American Stroke Association guidelines suggested using glucocorticoids for raised intracranial pressure and cerebral edema.^[11,18]^ The duration of oral anticoagulant treatment is usually between 3 and 12 months with a target international normalized ratio 2.0 to 3.0, but a longer duration may be required depending on the pathophysiology of CVT.^[11,41,52]^ Despite the controversy, antiphospholipid antibody syndrome and genetic thrombophilia may require continued life-long anticoagulation therapy because of a higher recurrence rate, and the benefits outweigh the risk of bleeding.^[11–14,41,51,52]^

In a recent randomized control trial, Connor and colleagues^[48]^ evaluated 114 children with CVT treated with rivaroxaban or standard anticoagulation therapy and observed favorable clinical outcomes with low risk of recurrence and fewer bleeding complications similar to other existing literature.^[41,48–52]^ In another study, Ferro and coworkers^[53]^ evaluated the safety and efficacy of dabigatran and warfarin in 120 patients from December 2016 to June 2018, with a follow-up of 25 weeks. This trial observed a low risk of recurrence and bleeding (about 1% and 3% in dabigatran and warfarin group, respectively), and recanalization rates were 60% and 67%, respectively, and recommended both dabigatran and warfarin safe and effective for preventing recurrent venous thrombosis in CVT.^[53]^

Furthermore, recent small non-randomized studies by Wasay et al^[54]^ and Nguyen et al^[55]^ also suggested that direct oral anticoagulants (DOACs), especially rivaroxaban and dabigatran, are safe and effective as warfarin in patients with CVT in reducing the bleeding risk and improving recanalisation rates. In addition to rivaroxaban and dabigatran, Lurkin and coworkers^[56]^ also found the efficacy of apixaban appears encouraging in CVT management despite variability in timing and dose of DOACs, similar to other study findings.^[57,58]^ The dosage of DOACs is variable ranging between 5 to 20 mg daily for rivaroxaban and 75 to 150 mg twice daily for dabigatran.^[54–59]^ Despite inconsistency in dosing, in a recent systemic review, Bose and colleagues^[60]^ observed the benefits of the DOACs over warfarin, including reduced dose adjustments and with no need to maintain a therapeutic international normalized ratio level similar to existing literature.^[48,53–60]^ Although DOAC do not require dose adjustment, several published papers observed that measuring plasma DOAC concentration is helpful in managing anticoagulated patients.^[61,62]^ Furthermore, clinicians and laboratory professionals should be aware that standard hemostatic parameters, especially the activity of antithrombin III, activated Protein C and S, and fibrinogen, may be affected by DOAC, which is why prothrombin time or activated partial thromboplastin time should not be performed as standalone tests to monitor the DOAC effect. The British Committee for Standards in Haematology recommended tests to assess DOAC effects are thrombin time and dilute thrombin time for dabigatran and anti-factor Xa activity for rivaroxaban, apixaban, and edoxaban.^[61–63]^

### 5.3. Endovascular intervention

In the late 1980s, endovascular treatment for CVT was first introduced. There are 2 distinct approaches; chemical thrombolysis and mechanical thrombectomy.^[14]^ Although favorable results for both are shown in case series, mechanical thrombectomy demonstrated better outcomes than thrombolysis.^[50–53,64]^ Use of mechanical thrombectomy is increasing, presumably because interventionalists use these techniques in ischemic stroke cases and have gained more experience.^[41,49,64,65]^

However, a recent RCT (Thrombolysis or anticoagulation for cerebral venous thrombosis; TO-ACT) trial by Coutinho and coworkers^[66]^ showed that endovascular treatment with standard medical care carries no significant difference in improving the clinical outcome of a severe form of CVT patients in comparison to standard medical care only. Nevertheless, endovascular treatment approaches to thrombolysis and thrombectomy are promising in the presence of large venous infarctions, brain herniation or intracranial hypertension.^[5,11,41,50–53,64–66]^

## 6. Prognosis and consequences

The treatment outcome of CVT is usually favorable with around 57% to 86% of the patients making a complete recovery, and mortality between 5.5% and 18%.^[5,7–11,45–49]^ However, approximately 6% to 10% of surviving patients have severe and permanent disability, and approximately 14% of patients require bed rest or hospital admission due to severe attacks of headaches.^[5,18,67]^ To date, there is no conclusive relationship between disease severity and treatment outcome; however, the cause of death is generally due to transtentorial herniation, status epilepticus or medical complications such as sepsis and pulmonary embolism.^[12,15–18,39,40,53]^ Several studies have presented potential predictors of poor outcome that include extreme age (infant and older age),^[6,9,10]^ altered mental status, rapid deterioration of consciousness (GCS < 9 on admission), coma and ICH,^[5,18,53]^ CNS infection,^[26–29]^ malignancy,^[30–32]^ thrombosis of the deep venous system,^[53,64–67]^ and hyperglycemia on admission.^[68]^

Approximately 12%, 14%, and 10% of the patients suffer from recurrence, different venous thrombosis, and seizures, respectively.^[37,67,69]^ Late seizures are more like to develop in those with a history of previous seizures, motor deficits, and supratentorial hemorrhagic lesions. Rarely intracranial hypertension might cause visual loss; thus, evaluation of the ophthalmological system should be performed in patients with papilloedema or altered vision.^[5,27,37,53,65–70]^

## 7. Conclusion

Cerebral venous sinus thrombosis is a rare but potentially fatal neurological condition that commonly affects young women of reproductive-age. It often remains underdiagnosed due to its nonspecific clinical presentation. A high degree of clinical suspicion is required as appropriate treatment at an early stage can improve the outcome. Low molecular weight heparin is recommended for acute treatment, while longer term treatment for 3 to 6 months is probably best undertaken with a direct oral anticoagulant.

## Author contributions

**Conceptualization:** Redoy Ranjan, Gie Ken-Dror, Pankaj Sharma.

**Data curation:** Redoy Ranjan.

**Resources:** Redoy Ranjan, Pankaj Sharma.

**Supervision:** Gie Ken-Dror, Pankaj Sharma.

**Validation:** Redoy Ranjan, Gie Ken-Dror, Pankaj Sharma.

**Visualization:** Redoy Ranjan.

**Writing – original draft:** Redoy Ranjan.

**Writing – review & editing:** Redoy Ranjan, Gie Ken-Dror, Pankaj Sharma.
